# Water Management Practices Affect Arsenic and Cadmium Accumulation in Rice Grains

**DOI:** 10.1155/2014/596438

**Published:** 2014-06-11

**Authors:** Liming Sun, Manman Zheng, Hongyan Liu, Shaobing Peng, Jianliang Huang, Kehui Cui, Lixiao Nie

**Affiliations:** National Key Laboratory of Crop Genetic Improvement, MOA Key Laboratory of Crop Ecophysiology and Farming System in the Middle Reaches of the Yangtze River, College of Plant Science and Technology, Huazhong Agricultural University, Wuhan, Hubei 430070, China

## Abstract

Cadmium (Cd) and arsenic (As) accumulation in rice grains is a great threat to its productivity, grain quality, and thus human health. Pot and field studies were carried out to unravel the effect of different water management practices (aerobic, aerobic-flooded, and flooded) on Cd and As accumulation in rice grains of two different varieties. In pot experiment, Cd or As was also added into the soil as treatment. Pots without Cd or As addition were maintained as control. Results indicated that water management practices significantly influenced the Cd and As concentration in rice grains and aerobic cultivation of rice furnished less As concentration in its grains. Nonetheless, Cd concentration in this treatment was higher than the grains of flooded rice. Likewise, in field study, aerobic and flooded rice cultivation recorded higher Cd and As concentration, respectively. However, growing of rice in aerobic-flooded conditions decreased the Cd concentration by 9.38 times on average basis as compared to aerobic rice. Furthermore, this treatment showed 28% less As concentration than that recorded in flooded rice cultivation. The results suggested that aerobic-flooded cultivation may be a promising strategy to reduce the Cd and As accumulations in rice grains simultaneously.

## 1. Introduction

Adverse effects of heavy metals on crops and increasing health hazards have attracted more and more attention in recent years. In China, nearly 10 billion ha of arable land has been contaminated by heavy metals mainly including cadmium, arsenic, lead, copper, nickel, zinc, mercury, and chromium [1]. In Zhujiang delta region of southern China, 28% of soil has been reported to be contaminated with heavy metals [2].

Rice (*Oryza sativa* L.), the staple food for more than half of the world's population, has been considered as a major source of Cd and As intake by humans in Asian countries, including Bangladesh, Japan, India, and China [3–6]. Both Cd and As are highly toxic heavy metals, which can be easily accumulated in rice crop posing significant risks to the public health through the food chain [7, 8]. Soil contamination by these two elements occurs mainly through anthropogenic activities, such as pesticide, fertilizer, herbicide application, mining, or irrigation with contaminated groundwater [9–11].

It is crucial to develop practical and effective strategies for reducing the amount and mobility of As and Cd in soil or to limit their uptake and accumulation in rice grains. Zhao et al. [12] and Kawasaki et al. [13] reported a great impact of water management practices on dynamics of Cd and As in soil and their subsequent uptake by rice. The facts of solubility of Cd varying with soil redox potential and the greater availability of As under anaerobic condition than aerobic environment support the possibility of reducing As and Cd accumulation in rice grain through water management [14, 15].

Although plenty of data are available on the effect of water management practices on Cd and As accumulation in rice grain, nevertheless, information is lacking regarding influence of aerobic-flooded rice system (dry direct-seeding culture) on Cd and As accumulation in rice grains. Present study was carried out in pots as well as in field aiming at investigating the Cd and As accumulations in rice grain under aerobic and flooded cultivations and to determine the effectiveness of aerobic-flooded treatment in minimizing the Cd and As accumulations in rice grains.

## 2. Materials and Methods

### 2.1. Pot Experiment

Pot experiment was carried out in greenhouse environment at Huazhong Agricultural University, Wuhan, Hubei Province, China (30°28′N 114°20′E). Five-liter plastic pots were filled with 5.0 kg air-dried, pulverized, and well-mixed soil taken from the top 25 cm layer of a field located at University Experimental Station. Physico-chemical properties of the soil are given in Table 1.

Each pot received 2.0 g urea and 2.0 g potassium dihydrogen phosphate one day prior to sowing followed by topdressing (1 g urea) at 30 days after sowing. Starter dose of fertilizer was promptly mixed into the soil and light irrigation was applied one day before sowing, to keep the soil moist. The treatments were Cd (2.5 mg Cd kg^−1^ soil as cadmium chloride) and As (5.0 mg As kg^−1^ soil in the form of arsenate (Na_2_HAsO_4_.7H_2_O, 7H_2_O)). The pots without Cd or As addition were considered as control.

In order to adapt to the aerobic condition, two aerobic rice varieties, Hanyou-3 and Lvhan-1, which have characteristics of drought tolerance and water saving were used. Hanyou-3 is a three-line hybrid rice variety, while Lvhan-1 is a recently developed inbred aerobic rice variety. To avoid the notion of shading, distance between pots was kept at 25 cm. Five uniform and healthy dry seeds were sown in each pot on 8th May 2012 and pots were kept saturated with water for one week after sowing to promote better stand establishment. One week after sowing, seedlings were thinned to two uniform seedlings per pot. Five pots from each treatment were kept under aerobic conditions, whereas the other five were kept under flooded conditions. The experiment was arranged in a completely randomized design with five replicates maintaining one pot per replication. The pots kept under aerobic condition were watered once after 1–3 days to keep the soil moisture tension from −15 to −25 kpa at 15 cm depth. For the flooded pots, 3–5 cm of standing water was kept in the pots throughout the experiment. Weeds were removed manually. Pesticides were sprayed 3-4 times to control insect damage. At maturity, plants were harvested and grains were threshed from panicles.

### 2.2. Field Experiment

A field experiment was conducted at the experimental station located in Dajin County, Hubei Province, China (29°51′N 115°33′E). Dajin County is one of the three main rice producing areas in Hubei Province. The same two rice varieties, Hanyou-3 and Lvhan-1, were used. The soil of the experimental site was gley and its physic-chemical properties are listed in Table 1.

Three water management treatments, namely, aerobic, aerobic-flooded, and flooded, were arranged in a randomized complete block design replicating four times with plot area of 30.0 m^2^ (6 m × 5 m). Aerobic and aerobic-flooded plots were dry-ploughed and harrowed during land preparation; soil was kept wet for one week after sowing to enhance better crop establishment. After one week, rainfed conditions were maintained in aerobic rice plots, whereas aerobic-flooded rice received flooding conditions from 5-leaf stage up to 2 weeks before harvest. Flooded plots were puddled and kept continuously flooded with 5–10 cm of water depth from transplanting until 2 weeks before harvest. For flooded rice, 25-day-old seedlings from wet bed nurseries were transplanted on 27th May 2012 at the rate of 3 seedlings per hill keeping the distance of 25.0 × 13.3 cm. For both aerobic and aerobic-flooded rice, dry seeds were directly sown in furrows manually with row spacing of 25 cm on 17th May 2012 using seed rate of 60 kg ha^−1^.

A standard fertilizer dose of 150 : 40 : 100 kg N : P : K ha^−1^ was applied in the form of urea, calcium superphosphate, and potassium chloride, respectively. All of the phosphorus, potassium, and 1/3rd of the N were applied as a starter basal dose (one day before sowing/transplanting), while residual N was split equally at middle tillering stage and panicle initiation stage. Pests, diseases, and weeds were intensively controlled. At maturity, plants were sampled from 0.5 m^2^ subplot, then panicles were manually threshed and filled spikelets were separated from unfilled spikelets.

### 2.3. As and Cd Analysis

Filled rice grains were ground to fine powder after oven drying at 70°C. Samples (0.5 g) were digested in 5 : 1 (v/v) HNO_3_/H_2_O_2_ (5 mL) in a microwave oven for 30 minutes (MLS 1200, Milestone, FKV, Italy) [16]. Cd and As concentrations in the digested samples were determined by inductively coupled plasma- (ICP-) optical emission spectroscopy (Vista-PRO, Varian, Inc., Palo Alto, CA,) and ICP-mass spectrometry (ICP-MS) (ELAN DRC-e, Perkin-Elmer Sciex, DE). Accuracy was evaluated by the use of a certified reference material (rice flour, NMIJ CRM 7502-a No.7 Cd Level II).

### 2.4. Statistical Analysis

Data were analyzed to confirm its variability following analysis of variance using Statistix 8.0. The differences between treatments were separated using least significance difference (LSD) test at 0.05 probability level.

## 3. Results and Discussion

### 3.1. The Cd and As Concentrations in Grains of Rice Grown under Aerobic and Flooded Cultivation in a Pot Experiment

**Figure 1:**
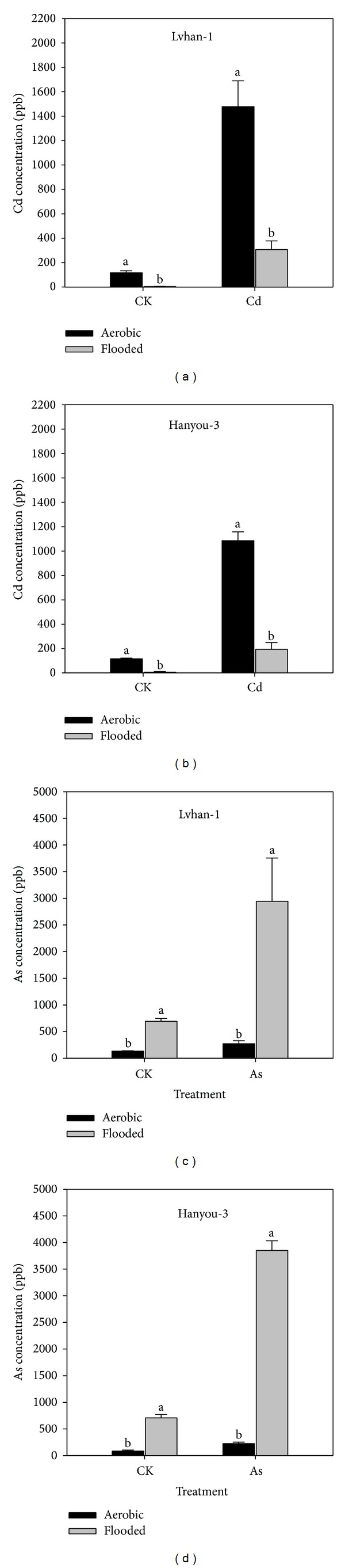
The Cd and As concentration in grains of Lvhan-1 and Hanyou-3 grown in the soil with additions of Cd, As, and without Cd or As addition (ck) under aerobic and flooded cultivation in a pot experiment. The rates of Cd and As were applied at 2.5 mg Cd kg^−1^ of soil as cadmium chloride and 5.0 mg As kg^−1^ of soil as cacodylic acid sodium salt, respectively. Error bars represent standard error of mean (SE).

Results showed that aerobically grown rice had significantly higher Cd and lower As accumulation compared with rice grown under flooded conditions (Figure 1). Applications of Cd at 2.5 mg Cd kg^−1^ soil or As at 5.0 mg As kg^−1^ soil markedly increased Cd or As concentrations in rice grains, respectively. The two rice varieties showed the similar trend. In the treatments without additions of Cd, Cd concentration in grains of Lvhan-1, and Hanyou-3 were 28 times and 18 times higher under aerobic condition than that under flooded condition, respectively. In the treatment without As addition, As concentrations in grains of Lvhan-1 and Hanyou-3 under flooded cultivation were 5.2 and 8.4 times as those under aerobic cultivation, respectively.

When Cd was applied at 2.5 mg Cd kg^−1^ soil, on average 421.5% of more Cd was accumulated in rice grains under aerobic conditions compared with that under flooded conditions (Figures 1(a) and 1(b)). In contrast, the average As concentration in rice grains of two rice varieties was 1297.2% higher under flooded cultivation than under aerobic cultivation, when As was added to the soil at 5.0 mg As kg^−1^ soil (Figures 1(c) and 1(d)).

### 3.2. The Cd and As Concentrations in Grains of Rice Grown under Aerobic, Flooded, and Aerobic-Flooded Cultivations in a Field Experiment

**Figure 2:**
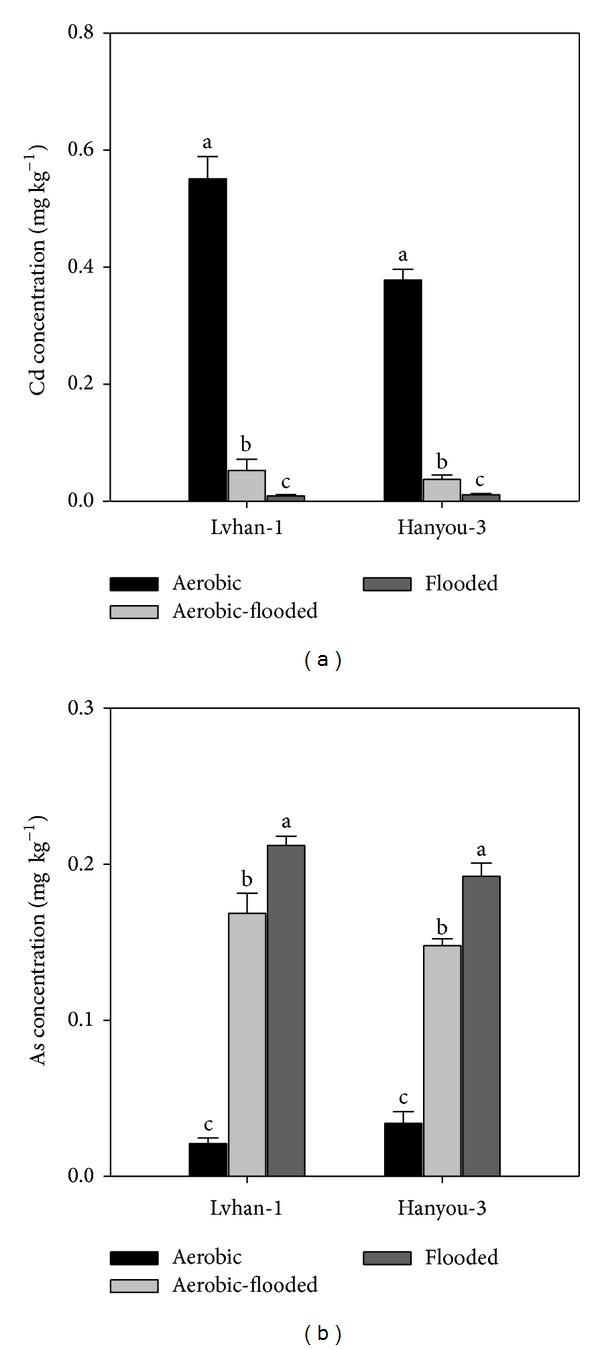
The Cd and As concentration in grains of Lvhan-1 and Hanyou-3 grown under three water management, aerobic, flooded, and aerobic-flooded cultivation in a field experiment. Error bars represent standard error of mean (SE).

More Cd was accumulated in grains of rice cultivated under aerobic conditions than under flooded conditions; however, As concentration was significantly lower in aerobic rice grains than flooded rice (Figure 2). The Cd concentrations in grains of Lvhan-1 and Hanyou-3 were 60.2 and 33.4 times higher under aerobic condition than under flooded condition, respectively (Figure 2(a)). On the contrary, Lvhan-1 and Hanyou-3 accumulated 910% and 466% more As in rice grains, respectively, under flooded than aerobic conditions (Figure 2(b)).

In the pot experiment, Cd concentration in rice grain was significantly higher; however, As concentration in grains was markedly lower under aerobic condition than that under flooded condition regardless of Cd or As addition (Figure 1). The results from pot experiment were confirmed by our field study. These findings are consistent with those of Arao et al. [17], who reported similar results in a pot experiment using Japanese rice varieties. Growing rice with less irrigation or under rainfed conditions led to less As concentration [18] and more Cd concentration in aerobic rice [13]. This might be attributed to decrease in the redox potential of the soil under flooded conditions that increases As concentration in the soil solution, whereas under aerobic conditions Cd concentration in the soil solution increases due to increase in redox potential of the soil [17]. Under aerobic conditions, As is usually present at very low concentrations in soil solution due to its strong adsorption by oxides/hydroxides of iron and aluminum [19]. It was documented that the higher Cd concentration in grains of rice cultivated aerobically than anaerobically was because of an increase in water-soluble Cd content, which was related with the increase in redox potential under aerobic condition [14].

### 3.3. Effect of Aerobic-Flooded Cultivation on Minimizing of Both Cd and As Accumulations in Rice Grains

Treatment of aerobic-flooded significantly decreased Cd and As concentrations in rice grains in comparison with the aerobic or flooded treatments, respectively (Figure 2). Under aerobic-flooded cultivation, the average Cd concentration in rice grains of two rice varieties was 9.38 times lower than that under aerobic cultivation (Figure 2(a)), while the average As concentration in rice grains was 28% lower than that of flooded cultivation (Figure 2(b)). Differences were also apparent regarding grain yield among different water management options and aerobic-flooded cultivation outperformed the aerobic cultivation (data not shown). Irrespective of cultivars, aerobic-flooded cultivation furnished grain yield of 8.3 t ha^−1^, which was statistically similar to flooded cultivation (8.7 t ha^−1^), but significantly higher than aerobically grown rice (7.6 t ha^−1^).

Previous research reported that Cd or As concentrations in rice might be mitigated by strategies, such as agronomic management practices, breeding, and genetic engineering [12, 13, 20]. Among agronomic management practices, water management was recommended as one of the most effective ways to reduce the As and Cd accumulation in rice plant. Previous studies reported that growing rice under aerobic conditions markedly reduced As accumulation in rice grains but significantly increased Cd accumulation [12, 17]. In our field study, aerobic-flooded cultivation appeared to be a promising strategy to reduce both Cd and As accumulations in rice grains simultaneously. Aerobic-flooded cultivation reduced Cd concentration in rice grains by an average of 9.38 times compared with aerobic cultivation and minimized average As concentration in rice grains by 28% in comparison with flooded cultivation (Figure 2).

Present study is of great concern because it justified the previous investigations in different locations under different edaphic and climatic conditions using different set of varieties in pot and field experiments. Although previous researchers have already reported different water management practices for minimizing Cd and As accumulation in rice grains, their rice establishment method was transplanting [17, 21, 22]. In present study, the establishment method opted for both of aerobic and aerobic-flooded cultivation was dry direct seeding. Furthermore, this study has explored the option of aerobic-flooded rice, which has proved effective for minimizing the accumulation of both heavy metals (Cd and As) in rice grains. The aerobic-flooded cultivation system possesses a vast application prospect in rice production areas because of the merits of water and labor saving and conducive to mechanization. However, the aspects of flooding durations and timing should be addressed in the future.

## 4. Conclusion

Rice grown aerobically obviously decreased As accumulation in rice grains but markedly increased Cd accumulation. Likewise, flooded rice has a demerit of enhanced As accumulation in rice grains. The present study suggested that a significant reduction of both Cd and As accumulations in rice grains could be achieved by aerobic-flooded cultivation of rice. However, the magnitude of decrease in Cd and As concentration in aerobic-flooded rice grains may be influenced by the duration of aerobic or flooded periods. This emphasizes the need for further research to get deeper insight regarding this area.

## Figures and Tables

**Table 1:** Physico-chemical properties of soils used for the pot and field experiments.

Parameter	Pot experiment	Field experiment	Ave. S.E.*
pH	5.8	5.0	0.08
Organic C (g kg^−1^)	13.9	17.7	0.96
Total N (g kg^−1^)	1.39	1.75	0.035
Olsen P (mg kg^−1^)	4.8	30.4	1.08
Available K (mg kg^−1^)	75	81	2.8
Arsenic (mg kg^−1^)	9.47	4.29	0.215
Cadmium (mg kg^−1^)	0.10	0.12	0.003

*Ave. S.E. is the mean of standard error (SE) of soil parameters in pot and field experiments.

## References

[1] Xinhua news agency (2013). *About 10 Billion Ha of Arable Land Was Contaminated By Heavy Metals in China*.

[2] Xue BN, Diao FC, Ren X (2013). *The Proportion of the Soil Contaminated By Heavy Metals Reached 28% in Zhujiang Delta Region of Southern China*.

[3] Tsukahara T, Ezaki T, Moriguchi J (2003). Rice as the most influential source of cadmium intake among general Japanese population. *Science of the Total Environment*.

[4] Mondal D, Polya DA (2008). Rice is a major exposure route for arsenic in Chakdaha block, Nadia district, West Bengal, India: a probabilistic risk assessment. *Applied Geochemistry*.

[5] Xu XY, McGrath SP, Meharg AA, Zhao FJ (2008). Growing rice aerobically markedly decreases arsenic accumulation. *Environmental Science and Technology*.

[6] Liang F, Li Y, Zhang G (2010). Total and speciated arsenic levels in rice from China. *Food Additives and Contaminants A Chemistry, Analysis, Control, Exposure and Risk Assessment*.

[7] Satarug S, Baker JR, Urbenjapol S (2003). A global perspective on cadmium pollution and toxicity in non-occupationally exposed population. *Toxicology Letters*.

[8] Rahman MA, Hasegawa H, Rahman MM, Mazid Miah MA, Tasmin A (2008). Arsenic accumulation in rice (*Oryza sativa L*.): Human exposure through food chain. *Ecotoxicology and Environmental Safety*.

[9] Takamatsu T, Aoki H, Yoshida T (1982). Determination of arsenate, arsenite, monomethylarsonate, and dimethylarsinate in soil polluted with arsenic. *Soil Science*.

[10] Sandalio LM, Dalurzo HC, Gómez M, Romero-Puertas MC, Del Río LA (2001). Cadmium-induced changes in the growth and oxidative metabolism of pea plants. *Journal of Experimental Botany*.

[11] Hu Z-Y, Zhu Y-G, Li M, Zhang L-G, Cao Z-H, Smith FA (2007). Sulfur (S)-induced enhancement of iron plaque formation in the rhizosphere reduces arsenic accumulation in rice (*Oryza sativa L*.) seedlings. *Environmental Pollution*.

[12] Zhao F-J, McGrath SP, Meharg AA (2010). Arsenic as a food chain contaminant: mechanisms of plant uptake and metabolism and mitigation strategies. *Annual Review of Plant Biology*.

[13] Kawasaki A, Arao T, Ishikawa S (2012). Reducing cadmium content of rice grains by means of flooding and a few problems. *Journal of the Food Hygienic Society of Japan*.

[14] Iimura K, Kitagishi K, Yamane I (1981). Heavy metals problems in paddy soils. *Heavy Metal Pollution in Soils of Japan*.

[15] Norton GJ, Pinson SRM, Alexander J (2012). Variation in grain arsenic assessed in a diverse panel of rice (*Oryza sativa L*.) grown in multiple sites. *New Phytologist*.

[16] Arao T, Takeda H, Nishihara E (2008). Reduction of cadmium translocation from roots to shoots in eggplant (*Solanum melongena*) by grafting onto *Solanum torvum* rootstock. *Soil Science and Plant Nutrition*.

[17] Arao T, Kawasaki A, Baba K, Mori S, Matsumoto S (2009). Effects of water management on cadmium and arsenic accumulation and dimethylarsinic acid concentrations in Japanese rice. *Environmental Science and Technology*.

[18] Rahaman S, Sinha AC (2013). Water regimes: an approach of mitigation arsenic in summer rice (*Oryza sativa L*.) under different topo sequences on arsenic-contaminated soils of Bengal delta. *Paddy and Water Environment*.

[19] Takahashi Y, Minamikawa R, Hattori KH, Kurishima K, Kihou N, Yuita K (2004). Arsenic behavior in paddy fields during the cycle of flooded and non-flooded periods. *Environmental Science and Technology*.

[20] Sebastian A, Prasad MNV (2014). Cadmium minimization in rice. A review. *Agronomy for Sustainable Development*.

[21] Hu P, Li Z, Yuan C (2013). Effect of water management on cadmium and arsenic accumulation by rice (*Oryza sativa L*.) with different metal accumulation capacities. *Journal of Soils and Sediments*.

[22] Hu P, Huang J, Ouyang Y (2013). Water management affects arsenic and cadmium accumulation in different rice cultivars. *Environmental Geochemistry and Health*.

